# Incorporating body mass index into esophageal manometry metrics and mean nocturnal baseline impedance for the evaluation of gastro-esophageal reflux disease

**DOI:** 10.1038/s41598-024-69253-2

**Published:** 2024-08-06

**Authors:** Meng-Han Tong, Ming-Jie Zhang, Li-Xia Wang, Zhi-Feng Zhang, Zhi-Jun Duan

**Affiliations:** Department of Gastroenterology, The First Affiliated Hospital of Dalian Medical University, Dalian, 116000 Liaoning China

**Keywords:** Gastro-esophageal reflux disease, High resolution manometry, EGJ contractile integral, Mean nocturnal baseline impedance, Decision curve analysis, Diseases, Gastroenterology

## Abstract

This study aims to enhance the effectiveness of high resolution manometry (HRM) and pH-impedance monitoring metrics in distinguishing between gastro-esophageal reflux disease (GERD) and non-GERD. A retrospective propensity score matching (PSM) study was conducted on 643 patients with GERD symptoms. PSM matched 134 GERD patients with 134 non-GERD controls. Body mass index (BMI), intra-esophageal pressure (IEP) and intra-gastric pressure (IGP) were significantly higher in the GERD group compared to the non-GERD group. BMI was correlated with IEP and IGP positively. IGP was positively correlated with esophagogastric (EGJ) pressure (EGJ-P) in participants with EGJ type 1 and 2, but not in participants with EGJ type 3. BMI was correlated with distal MNBI negatively. Logistic regression showed BMI as an independent risk factor for GERD. Receiver operating characteristic curve (ROC) and decision curve analysis (DCA) showed that BMI adjusted EGJ contractile integral (EGJ-CI) and BMI adjusted MNBI were superior to the corresponding original ones in predicting GERD susceptibility. According to the findings, BMI and IGP are the main factors contributing to the development of GERD. BMI affects IEP through the adaptive response of EGJ-P to IGP. Incorporating BMI into the calculations of EGJ-CI and MNBI can improve their ability in predicting GERD susceptibility.

## Introduction

Gastro-esophageal reflux disease (GERD) is a common gastrointestinal condition worldwide. The pooled global prevalence of GERD is estimated to be 13.3%, which is derived from a meta-analysis^1^. Complications of GERD encompass esophagitis, peptic esophageal stricture, Barrett esophagus (BE) and subsequent esophageal adenocarcinoma^2,3^. Pathophysiology of GERD includes esophagogastric junction (EGJ) barrier dysfunction, transient LES relaxations (TLESRs), weakened esophageal body motility and increased gastro-esophageal pressure gradient (GEG)^4–7^. Esophageal high resolution manometry (HRM) has been popularly used to assess esophageal motility worldwide. Metrics of esophageal HRM have the advantage to stratify GERD severity and provide additional diagnostic values to GERD^8^. EGJ contractile integral (EGJ-CI) measured with distal contractile integral (DCI) tool is advocated by Chicago Classification version 4.0 to evaluate EGJ barrier function^9^. EGJ-CI assesses contractility of lower esophageal sphincter (LES) and crural diaphragm (CD) simultaneously, and EGJ length is also taken into the calculation of EGJ-CI. However, anti-reflux pressure barrier (APB) is not only determined by LES and CD, but also affected by GEG^6^. Therefore, novel metrics combining contractility of LES and CD, GEG and EGJ length might represent the realistic APB.

Chicago Classification version 4.0 recommends EGJ-CI to be referenced to intra-gastric pressure (IGP) instead of atmospheric pressure^9^. This method incorporates IGP into EGJ-CI calculation. Obesity is positively associated with GERD symptoms and esophageal acid burdens^10^. Moreover, IGP is positively associated with body mass index (BMI)^11^. So EGJ-CI encompassing the effect of IGP on APB has its advantage in predicting GERD susceptibility especially in the condition of obesity. However, GEG is the value of IGP minus intra-esophageal pressure (IEP), IGP cannot represent GEG completely. Additionally, the change of IEP is dependent on different conditions. IEP during expiration is positively correlated with BMI in the general population^12^. On the contrary, decreased IEP contributes to GERD development in chronic pulmonary obstructive disease (COPD) patients^13^. So EGJ-CI encompassing the effect of GEG instead of the effect of IGP might reflect the real condition of APB and increase its ability in predicting GERD susceptibility. Furthermore, obesity is positively correlated with TLESRs which plays a role in increasing GERD susceptibility^14^. TLESRs can weaken APB, however, TLESRs cannot be routinely measured in clinical settings and incorporated into the calculation of EGJ-CI. Therefore, incorporating BMI into the calculation of EGJ-CI might be appropriate and increase its ability to stratify and diagnose GERD. Different from esophageal HRM, 24-h pH-impedance monitoring tests can measure esophageal mucosal integrity with esophageal baseline impedance^15^. A previous study has demonstrated that distal esophageal mean nocturnal baseline impedance (MNBI) is associated with esophageal acid exposure time (AET) and has a diagnostic value to discriminate GERD from non-GERD^16^. Moreover, MNBI is negatively correlated with BMI in patients with typical GERD symptoms^17^. So incorporating BMI into calculation of MNBI might also increase its ability to discriminate GERD.

This study aims to (1) study GERD pathophysiology with esophageal HRM and MNBI, (2) evaluate whether EGJ-CI by the reference to intra-gastric pressure is superior to EGJ-CI by the reference to atmospheric pressure, (3) assess whether EGJ-CI adjusted with BMI is superior to the original one, (4) evaluate whether MNBI adjusted with BMI is superior to the original one, (5) find novel metrics combining contractility of LES and CD, GEG and EGJ length, and assess their ability to discriminate GERD from non-GERD.

## Methods

### Study population and design

Data of consecutive patients who were with GERD symptoms was retrospectively retrieved from the Gastrointestinal Motility Center of the First Affiliated Hospital of Dalian Medical University from January 2015 to December 2023. Moreover, in order to maintain data consistency among patients, we only included data on the same HRM system (Medkinetic Incorporated, Ningbo, China) and data on the same 24-h pH-impedance monitoring system (OMOM system). Moreover, all patients were of Chinese race but not Caucasian or other races. Inclusion criteria: (1) Age was more than 18 years. (2) All had undergone 24-h pH-impedance monitoring and concurrent esophageal HRM. (3) All esophageal function tests were performed without proton pump inhibitor (PPI) or prokinetic drugs within two weeks. (4) All had a gastroscopy prior to esophageal function test. (5) Information on age, gender, weight and height was complete. (6) Definite GERD or non-GERD patients based on 24-h pH monitoring were included. Exclusion criteria: (1) Patients had gastrointestinal operations and tumors. (2) HRM showed achalasia, EGJ obstruction, high contractile esophagus and distal esophageal spasm according to Chicago Classification version 4.0. (3) Gastroscopy showed esophageal stricture. (4) 24-h pH-impedance monitoring and concurrent esophageal HRM were of poor quality. (5) 24-h pH monitoring provided borderline results. The retrospective study was approved by the Ethic Committee of the First Affiliated Hospital of Dalian Medical University (PJ-KS-KY-2024-131). Waiver of informed consent was validated by the Ethic Committee of the First Affiliated Hospital of Dalian Medical University. The retrospective study was performed in accordance with the Declaration of Helsinki.

### 24-h pH-impedance monitoring studies

24-h pH-impedance monitoring tests were performed with multichannel intraluminal impedance-pH monitoring OMOM system (OMOM, Jinshan Science and Technology, Chongqing, China). An experienced nurse placed the pH sensor of a monitoring catheter at 5 cm above the upper edge of the LES. Impedance electrodes were placed at 3cm (Z6), 5cm (Z5), 7cm (Z4), 9cm (Z3), 15cm (Z2) and 17cm (Z1) above the LES respectively. Patients were instructed to write a monitoring diary accordingly. No restriction to the regular activities and regular meals was used in this study. However, alcohol, beverages, acidic food (e.g. vinegar), boiled and icy water were avoided during 24-h pH-impedance monitoring tests. 24 h later, patients would return to the Gastrointestinal Motility Center, and the monitoring catheters would be removed. The monitoring catheters were connected with a dedicated data analysis software (Jinshan Science and Technology, Chongqing, China) for data collection and analysis. Data of total reflux episodes, AET, DeMeester score (DMS) and MNBI of six channels (Z1–Z6) were collected in this study. MNBI was calculated according to previous studies^16,17^. AET < 4% was recognized as negative, AET > 6% was recognized as positive, and AET between 4 and 6% was recognized as borderline according to Lyon consensus^18^. Moreover, BMI was calculated with the method recommended by the World Health Organization (WHO)^19^. Because BMI more than 25 kg/m^2^ was considered to be overweight or obese^19^, we used BMI of participants divided by 25 kg/m^2^ to standardize BMI. Consequently, MNBIZ5 and MNBIZ6 were divided by standardized BMI to obtain BMI adjusted MNBIZ5 (adMNBIZ5) and BMI adjusted MNBIZ6 (adMNBIZ6). Through this method, BMI was incorporated into the calculation of MNBI.

### Esophageal HRM

Gastrointestinal pressure measuring system GAP-36A with a 24-channel water-perfused catheter (Medkinetic Incorporated, Ningbo, China) was employed to perform esophageal HRM. Patients were free of PPI and prokinetic drugs for at least two weeks prior to esophageal HRM. Esophageal HRM was performed in fasting condition of patients. On the examination day, an experienced nurse inserted a pressure measuring catheter trans-nasally and assured the catheter in the stomach. The rest pressure of UES and LES was recorded for at least 30 s. Afterwards, patients would swallow 5 ml water at room temperature for 10 times. The interval between adjacent swallows was 30 s. MedView360 software provided by the GAP-36A system was used for analysis. All esophageal HRM analyses were according to the Chicago Classification version 4.0^9^. HRM metrics representing EGJ anti-reflux functions and esophageal body peristalsis were included into analyses. Because integrated relaxation pressure (IRP) represented the relaxation function of EGJ but not EGJ anti-reflux functions, IRP was not included into our analyses. Distal esophageal spasm was excluded from our study, so distal latency (DL) was not included into our analyses either. Additionally, some novel metrics related to EGJ anti-reflux functions were measured by the reference to atmospheric pressure during the baseline recording in supine position. We measured mean IGP at 2 cm below the EGJ lower border in 3 respiratory cycles as modified from a previous study^6^. The mean IEP was measured at 2 cm above the EGJ upper border in 3 respiratory cycles. GEG was calculated as IGP minus IEP. The mean rest pressure of EGJ in 3 respiratory cycles was defined as EGJ rest pressure (EGJ-P). EGJ retention pressure (EGJ-RP) was calculated as EGJ-P minus GEG^6^. EGJ-RP integral (EGJ-RI) was calculated as EGJ-RP multiplied by EGJ length. EGJ-CI was calculated by the reference to atmospheric pressure (EGJ-CIA) or intra-gastric pressure (EGJ-CIG) using the intelligent mouse tool specific for EGJ-CI calculation (Medkinetic Incorporated, Ningbo, China). EGJ-CI, EGJ-RP and EGJ-RI were divided by standardized BMI to obtain BMI adjusted EGJ-CI (adEGJ-CI), BMI adjusted EGJ-RP (adEGJ-RP) and BMI adjusted EGJ-RI (adEGJ-RI). Through this method, BMI was incorporated into the calculations of EGJ HRM metrics.

### Statistical analysis

SPSS 26.0 was used for propensity score matching (PSM), SPSS 26.0 and Stata 14.0 were used for statistical analysis. R (version 4.3.2) software was used in the depiction of statistical figures. Age and gender were used to calculate propensity scores. Nearest-neighbor PSM was performed with a 1:1 ratio and a match tolerance of 0.02. Median, 25th percentile and 75 percentile were used to express quantitative data. The Wilcoxon Rank Sum test was utilized to compare differences of quantitative variables between the groups. Pearson’s Chi square test was used to compare differences of qualitative variables between the groups. Spearman correlation analysis was used to evaluate correlations among interested variables. Logistic regression analysis was used to perform multivariate analysis. Receiver operating characteristic curve (ROC) was employed to evaluate HRM metrics and MNBI in predicting GERD susceptibility. Decision curve analysis (DCA) was used to assess the clinical net benefit of metrics in predicting GERD susceptibility^20^. Values of *P* < 0.05 were considered statistically significant.

## Results

### Study population and PSM

**Figure 1:**
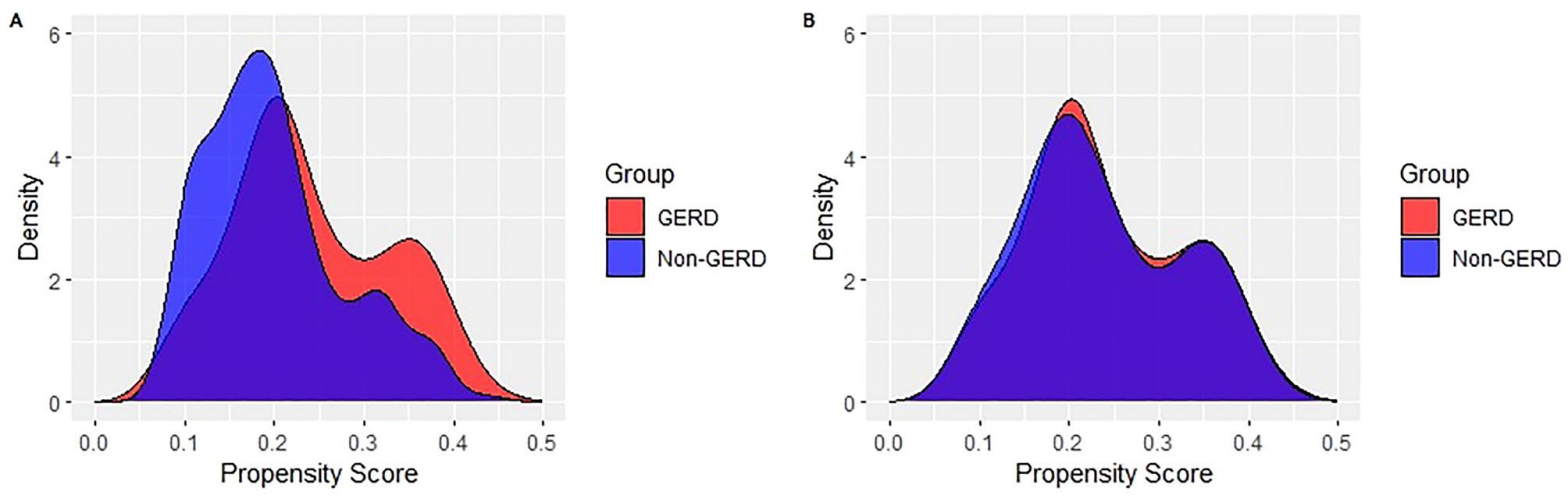
The distribution of propensity scores before and after PSM. (**A**) The distribution of propensity scores between the GERD group and the Non-GERD group before PSM. (**B**) The distribution of propensity scores between the GERD group and the Non-GERD group after PSM. *PSM* propensity score matching.

A total of 643 records with 134 GERD patients and 509 non-GERD controls were retrieved according to the inclusion criteria and exclusion criteria. PSM matched 134 GERD patients with 134 non-GERD controls successfully. The distribution of propensity scores between GERD patients and non-GERD patients overlapped almost completely after PSM. The distribution of propensity scores before and after PSM was illustrated in Fig. 1. Age and gender were comparable between the groups after PSM as demonstrated in Table S1 of Supplementary materials. The distribution of age according to gender before and after PSM was illustrated in Fig. S1 of Supplementary materials.

### Esophageal HRM metrics

**Figure 2:**
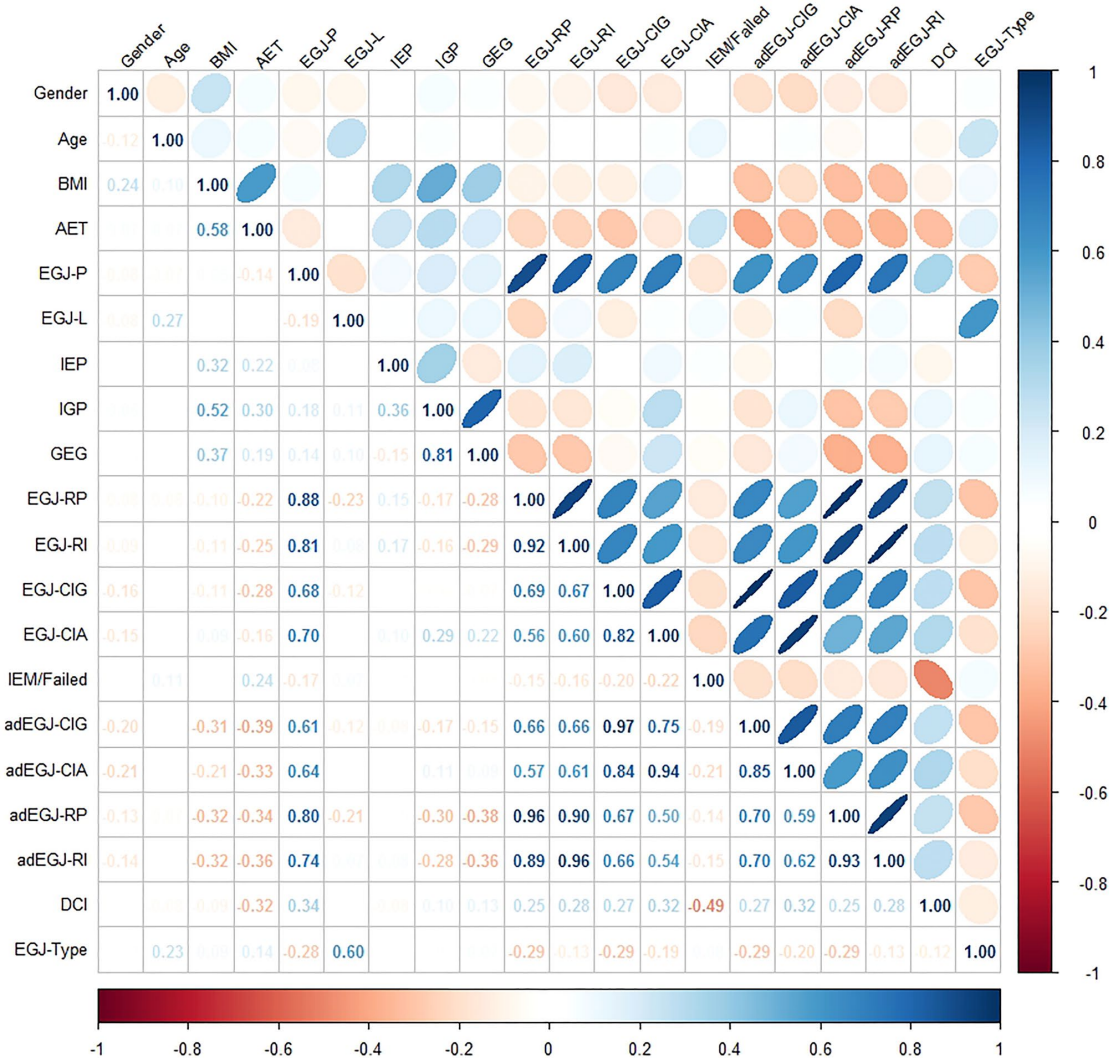
Spearman correlation analysis of demographics and esophageal HRM metrics. *BMI* body mass index, *AET* acid exposure time, *EGJ*-*P* EGJ rest pressure, *EGJ*-*L* EGJ length, *DCI* distal contractile integral, *IEM* ineffective esophageal motility, *IEP* intra-esophageal pressure, *IGP* intra-gastric pressure, *GEG* gastric-esophageal gradient, *EGJ*-*RP* EGJ retention pressure, *EGJ*-*RI* EGJ-RP integral, *EGJ*-*CIA* EGJ contractile integral (EGJ-CI) by the reference to atmospheric pressure, *EGJ*-*CIG* EGJ-CI by the reference to intra-gastric pressure, *adEGJ*-*CIA* EGJ-CIA adjusted with BMI, *adEGJ*-*CIG* EGJ-CIG adjusted with BMI, *adEGJ*-*RP* EGJ-RP adjusted with BMI, *adEGJ*-*RI* EGJ-RI adjusted with BMI.

**Figure 3:**
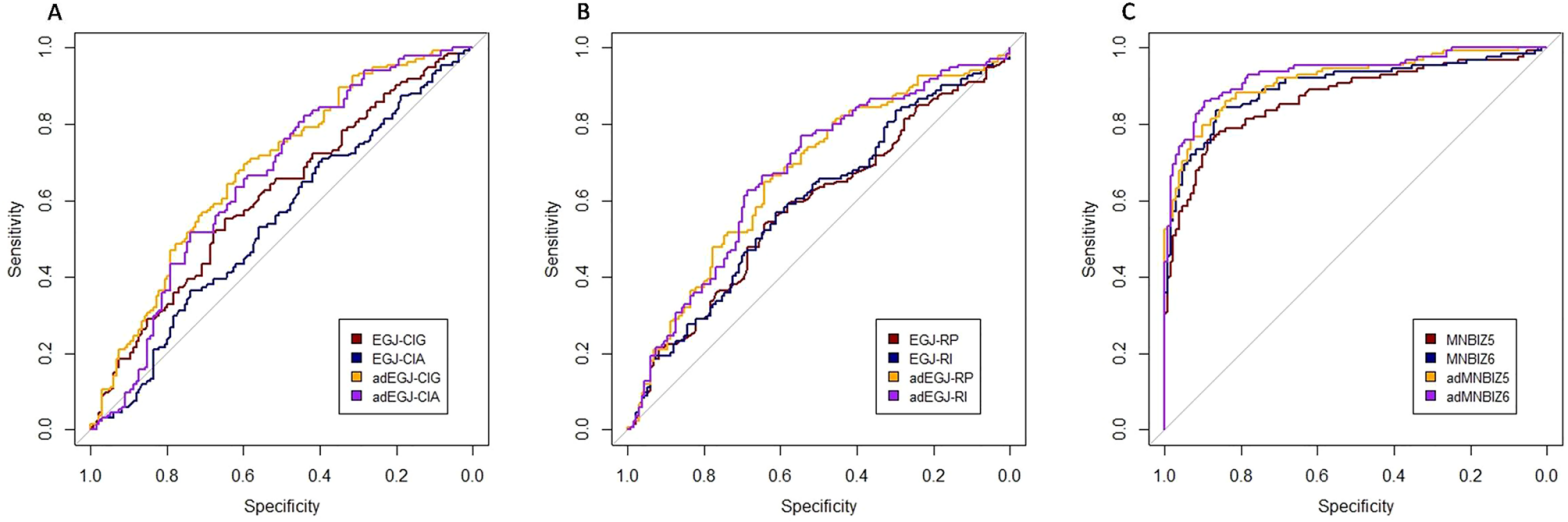
ROC of esophageal HRM metrics and MNBI in predicting GERD susceptibility. (**A**) ROC of EGJ-CIG, EGJ-CIA, adEGJ-CIG and adEGJ-CIA in predicting GERD susceptibility. AUC of EGJ-CIG: 0.61 (95% CI 0.54–0.68). AUC of EGJ-CIA: 0.54 (95% CI 0.47–0.61). AUC of adEGJ-CIG: 0.68 (95% CI 0.62–0.74). AUC of adEGJ-CIA: 0.65 (95% CI 0.59–0.72). (**B**) ROC of EGJ-RP, EGJ-RI, adEGJ-RP and adEGJ-RI in predicting GERD susceptibility. AUC of EGJ-RP: 0.58 (95% CI 0.51–0.65). AUC of EGJ-RI: 0.59 (95% CI 0.53–0.66). AUC of adEGJ-RP: 0.67 (95% CI 0.60–0.73). AUC of adEGJ-RI: 0.67 (95% CI 0.61–0.74). (**C**) ROC of MNBIZ5, MNBIZ6, adMNBIZ5 and adMNBIZ6 in predicting GERD susceptibility. AUC of MNBIZ5: 0.87 (95% CI 0.83–0.92). AUC of MNBIZ6: 0.90 (95% CI 0.86–0.94). AUC of adMNBIZ5: 0.92 (95% CI 0.88–0.95). AUC of adMNBIZ6: 0.93 (95% CI 0.90–0.96). *EGJ*-*CIG* EGJ contractile integral (EGJ-CI) by the reference to intra-gastric pressure, *EGJ*-*CIA* EGJ-CI by the reference to atmospheric pressure, *adEGJ*-*CIG* EGJ-CIG adjusted with BMI, *adEGJ*-*CIA* EGJ-CIA adjusted with BMI, *MNBIZ5* mean nocturnal baseline impedance channel 5, *MNBIZ6* mean nocturnal baseline impedance channel 6, *adMNBIZ5* MNBIZ5 adjusted with BMI, *adMNBIZ6* MNBIZ6 adjusted with BMI, *AUC* area under curve, *95% CI* 95% confidence interval.

BMI, IEP, IGP and GEG were significantly higher in the GERD group compared with those in the non-GERD group. EGJ-RP, EGJ-RI, EGJ-CIG, adEGJ-RP, adEGJ-RI, BMI adjusted EGJ-CIA (adEGJ-CIA) and BMI adjusted EGJ-CIG (adEGJ-CIG) were significantly lower in the GERD group compared with those in the non-GERD group. EGJ-CIA was lower in the GERD group compared with that in the non-GERD group but not significant. Direct comparison was illustrated in Table 1. Spearman correlation analysis showed that BMI was correlated with IEP (r = 0.32, *P* < 0.0001), IGP (r = 0.52, *P* < 0.0001) and GEG (r = 0.37, *P* < 0.0001) positively and significantly. BMI was correlated with EGJ-RP (r = − 0.10, *P* = 0.10), EGJ-RI (r = − 0.11, *P* = 0.60) and EGJ-CIG (r = − 0.11, *P* = 0.14) negatively but not significantly. EGJ-CIA was correlated with IGP (r = 0.29, *P* < 0.0001) and GEG (r = 0.22, *P* < 0.001) positively and significantly. IGP was correlated with EGJ-P (r = 0.18, *P* < 0.01) and IEP (r = 0.36, *P* < 0.0001) positively and significantly. In subgroup analysis, IGP was positively correlated with EGJ-P in participants with EGJ type 1 (r = 0.24, *P* < 0.01) and type 2 (r = 0.30, *P* = 0.02). IGP was not correlated with EGJ-P in participants with EGJ type 3 (r = 0.11, *P* = 0.45). Spearman correlation analysis among demographics and esophageal HRM metrics was illustrated in Fig. 2. ROC showed that EGJ-CIG was superior to EGJ-CIA in discriminating GERD from non-GERD. EGJ-RP and EGJ-RI were not superior to EGJ-CIG in discriminating GERD from non-GERD. adEGJ-CIG is most efficient in discriminating GERD from non-GERD. ROC was illustrated in Fig. 3A and B. Detailed comparisons of ROC among esophageal HRM metrics were illustrated in Table S2 of Supplementary materials. The optimal discriminating threshold of EGJ-CIG and adEGJ-CIG was 22 mmHg.cm (sensitivity = 0.65, specificity = 0.55) and 28 mmHg.cm (sensitivity = 0.62, specificity = 0.67) respectively in our study. When we used the value of 25 mmHg.cm recommended by Chicago classification version 4.0 to discriminate GERD^9^, the sensitivity of EGJ-CIG was 0.60 and the specificity of EGJ-CIG was 0.57 in our study.

**Table 1:** Demographics, esophageal HRM metrics and pH-impedance monitoring metrics between the groups.

	GERD	Non-GERD	*P*
Age (years)*	60 (46–68)	60 (47–67)	0.93
Gender (male/female)	71/63	66/68	0.54
BMI (kg/m^2^)*	24.97 (21.99–27.34)	19.98 (18.37–21.68)	< 0.0001
AET (total %)*	10.20 (8.00–14.30)	0.50 (0.10–1.30)	< 0.0001
DMS*	37.45 (27.60–49.70)	2.70 (1.10–5.20)	< 0.0001
Reflux episode*	43.00 (28.00–63.00)	18.50 (9.00–32.00)	< 0.0001
MNBIZ1 (ohms)*	1801.00 (1253.00–2192.00)	1779.00 (1409.00–2231.00)	0.32
MNBIZ2 (ohms)*	1677.00 (1120.00–2073.00)	1678.00 (1313.00–2157.00)	0.11
MNBIZ3 (ohms)*	1230.50 (977.50–1841.00)	2011.00 (1558.00–2510.00)	< 0.0001
MNBIZ4 (ohms)*	1183.00 (859.50–1839.50)	2149.00 (1681.00–2599.00)	< 0.0001
MNBIZ5 (ohms)*	1047.50 (712.00–1448.50)	2157.00 (1709.00–2522.00)	< 0.0001
MNBIZ6 (ohms)*	886.50 (609.00–1348.00)	2053.00 (1651.00–2395.00)	< 0.0001
adMNBIZ5 (ohms)*	1045.70 (714.86–1470.34)	2733.68 (2052.38–3262.40)	< 0.0001
adMNBIZ6 (ohms)*	927.42 (571.27–1290.80)	2597.64 (2034.35–3120.17)	< 0.0001
DCI (mmHg.s.cm)*	1007.60 (562.10–1593.8)	1594.00 (876.60–2665.30)	< 0.0001
IEM/failed peristalsis (yes/no)	24/110	11/123	0.02
EGJ-type (I/II/III)	73/30/31	84/31/19	0.16
EGJ-P (mmHg)*	12.80 (9.00–20.00)	14.00 (10.00–19.00)	0.28
EGJ-L (cm)*	3.34 (2.80–3.93)	3.49 (2.82–4.00)	0.80
IEP (mmHg)*	0.05 (− 0.6–0.90)	− 0.40 (− 1.30–0.20)	< 0.0001
IGP (mmHg)*	5.25 (2.90–7.60)	3.00 (1.10–5.50)	< 0.0001
GEG (mmHg)*	5.10 (3.00–7.00)	4.15 (1.80–6.20)	0.01
EGJ-RP (mmHg)*	7.60 (4.10–12.80)	9.85 (5.8–14.70)	0.01
EGJ-RI (mmHg.cm)*	24.28 (12.58–41.16)	33.15 (18.27–48.48)	< 0.01
EGJ-CIA (mmHg.cm)*	36.50 (23.50–54.80)	42.85 (27.30–58.50)	0.26
EGJ-CIG (mmHg.cm)*	20.60 (8.90–37.00)	27.60 (15.50–44.30)	< 0.01
adEGJ-CIA (mmHg.cm)*	34.74 (24.81–52.08)	51.26 (32.95–74.23)	< 0.0001
adEGJ-CIG (mmHg.cm)*	20.01 (9.61–34.86)	35.12 (20.01–60.02)	< 0.0001
adEGJ-RP (mmHg)*	7.71 (4.15–12.39)	12.45 (7.42–19.13)	< 0.0001
adEGJ-RI (mmHg.cm)*	26.12 (13.97–38.57)	40.59 (22.38–60.92)	< 0.0001

*BMI* body mass index, *AET* acid exposure time, *DMS* DeMeester score, *MNBIZ1*-*Z6* mean nocturnal baseline impedance channel Z1-Z6, *adMNBIZ5* MNBIZ5 adjusted with BMI, *adMNBIZ6* MNBIZ6 adjusted with BMI, *DCI* distal contractile integral, *IEM* ineffective esophageal motility, *EGJ*-*P* EGJ rest pressure, *EGJ*-*L* EGJ length, *IEP* intra-esophageal pressure, *IGP* intra-gastric pressure, *GEG* gastro-esophageal gradient, *EGJ*-*RP* EGJ retention pressure, *EGJ*-*RI EGJ*-*RP* integral, *EGJ*-*CIA* EGJ contractile integral (EGJ-CI) by the reference to atmospheric pressure, *EGJ*-*CIG* EGJ-CI by the reference to intra-gastric pressure, *adEGJ*-*CIA* EGJ-CIA adjusted with BMI, *adEGJ*-*CIG* EGJ-CIG adjusted with BMI, *adEGJ*-*RP* EGJ-RP adjusted with BMI, *adEGJ*-*RI* EGJ-RI adjusted with BMI.

*Data was expressed with Median, 25th percentile and 75 percentile.

### 24-h pH-impedance monitoring metrics

AET, DMS and reflux episode were significantly higher in the GERD group compared with those in the non-GERD group. MNBIZ3, MNBIZ4, MNBIZ5 and MNBIZ6 were significantly lower in the GERD group compared with those in the non-GERD group. MNBIZ1 and MNBIZ2 were not significantly different between the groups. Direct comparison was illustrated in Table 1. Spearman correlation analysis showed that BMI was correlated with AET (r = 0.58, *P* < 0.0001), MNBIZ3 (r = − 0.37, *P* < 0.0001), MNBIZ4 (r = − 0.42, *P* < 0.0001), MNBIZ5 (r = − 0.46, *P* < 0.0001) and MNBIZ6 (r = − 0.48, *P* < 0.0001). Spearman correlation analysis among BMI and MNBI was illustrated in Fig. S2 of Supplementary materials. ROC showed that MNBIZ6 was superior to MNBIZ5 in discriminating GERD from non-GERD. adMNBIZ6 is most efficient in discriminating GERD from non-GERD. ROC of different metrics was illustrated in Fig. 3C. Detailed comparisons among MNBI and adMNBI were illustrated in Table S3 of Supplementary materials. The optimal diagnosing threshold of MNBIZ6 and adMNBIZ6 was 1458 ohms (sensitivity = 0.86, specificity = 0.84) and 1623 ohms (sensitivity = 0.89, specificity = 0.86) respectively in our study. When we used the value of 1500 ohms recommended by Lyon consensus to diagnose GERD^18^, the sensitivity of MNBIZ6 was 0.84 and the specificity of MNBIZ6 was 0.83 in our study.

### Correlation between esophageal HRM metrics and 24-h pH-impedance monitoring metrics

Spearman correlation analysis showed that AET was negatively associated with EGJ-P (r = − 0.14, *P* = 0.02), EGJ-RP (r = − 0.22, *P* < 0.001), EGJ-RI (r = − 0.25, *P* < 0.0001), EGJ-CIG (r = − 0.28, *P* < 0.0001), EGJ-CIA (r = − 0.16, *P* < 0.01), adEGJ-CIG (r = − 0.39, *P* < 0.0001), adEGJ-CIA (r = − 0.33, *P* < 0.0001), adEGJ-RP (r = − 0.34, *P* < 0.0001), adEGJ-RI (r = − 0.36, *P* < 0.0001) and DCI (r = − 0.32, *P* < 0.0001). AET was positively associated with IEP (r = 0.22, *P* < 0.001), IGP (r = 0.30, *P* < 0.0001), GEG (r = 0.19, *P* < 0.01) and EGJ-type (r = 0.14, *P* = 0.02). AET was not significantly associated with EGJ-L (r = 0.01, *P* = 0.83).

Reflux episode was negatively associated with EGJ-P (r = − 0.14, *P* = 0.02), EGJ-RP (r = − 0.20, *P* < 0.01), EGJ-RI (r = − 0.21, *P* < 0.001), EGJ-CIG (r = − 0.27, *P* < 0.0001), EGJ-CIA (r = − 0.19, *P* < 0.01), adEGJ-CIG (r = − 0.33, *P* < 0.0001), adEGJ-CIA (r = − 0.30, *P* < 0.0001), adEGJ-RP (r = − 0.27, *P* < 0.0001), adEGJ-RI (r = − 0.28, *P* < 0.0001) and DCI (r = − 0.22, *P* < 0.001). Reflux episode was positively associated with IEP (r = 0.15, *P* = 0.01) and IGP (r = 0.21, *P* < 0.001). Reflux episode was not significantly associated with EGJ-L (r = − 0.05, *P* = 0.41), GEG (r = 0.11, *P* = 0.06) and EGJ-type (r = 0.10, *P* = 0.13).

MNBIZ5 was positively associated with EGJ-CIG (r = 0.12, *P* < 0.05), adEGJ-CIG (r = 0.20, *P* < 0.01), adEGJ-RP (r = 0.19, *P* < 0.01), adEGJ-RI (r = 0.18, *P* < 0.01) and DCI (r = 0.25, *P* < 0.0001). MNBIZ5 was negatively associated with IEP (r = − 0.18, *P* < 0.01), IGP (r = − 0.29, *P* < 0.0001), GEG (r = − 0.17, *P* < 0.01) and EGJ-type (r = − 0.18, *P* < 0.01). MNBIZ5 was not significantly associated with EGJ-P (r = 0.03, *P* = 0.61), EGJ-L (r = − 0.05, *P* = 0.45), EGJ-RP (r = 0.10, *P* = 0.10), EGJ-RI (r = 0.10, *P* = 0.10), EGJ-CIA (r = − 0.01, *P* = 0.91) and adEGJ-CIA (r = 0.11, *P* = 0.08).

MNBIZ6 was positively associated with EGJ-RP (r = 0.15, *P* = 0.02), EGJ-RI (r = 0.16, *P* = 0.01), EGJ-CIG (r = 0.17, *P* = 0.01), adEGJ-CIG (r = 0.25, *P* < 0.0001), adEGJ-CIA (r = 0.16, *P* = 0.01), adEGJ-RP (r = 0.25, *P* < 0.0001), adEGJ-RI (r = 0.25, *P* < 0.0001) and DCI (r = 0.28, *P* < 0.0001). MNBIZ6 was negatively associated with IEP (r = − 0.21, *P* < 0.001), IGP (r = − 0.33, *P* < 0.0001), GEG (r = − 0.21, *P* < 0.0001) and EGJ-type (r = − 0.19, *P* < 0.01). MNBIZ6 was not significantly associated with EGJ-P (r = 0.06, *P* = 0.32), EGJ-L (r = − 0.04, *P* = 0.57) and EGJ-CIA (r = 0.03, *P* = 0.59).

adMNBIZ5 was positively associated with EGJ-CIG (r = 0.13, *P* = 0.03), adEGJ-CIG (r = 0.26, *P* < 0.0001), adEGJ-CIA (r = 0.15, *P* = 0.01), adEGJ-RP (r = 0.26, *P* < 0.0001), adEGJ-RI (r = 0.25, *P* < 0.001) and DCI (r = 0.23, *P* < 0.001). adMNBIZ5 was negatively associated with IEP (r = − 0.26, *P* < 0.0001), IGP (r = − 0.40, *P* < 0.0001), GEG (r = − 0.25, *P* < 0.0001) and EGJ-type (r = − 0.17, *P* < 0.01). adMNBIZ5 was not significantly associated with EGJ-P (r = − 0.01, *P* = 0.92), EGJ-L (r = − 0.04, *P* = 0.47), EGJ-RP (r = 0.11, *P* = 0.08), EGJ-RI (r = 0.11, *P* = 0.07) and EGJ-CIA (r = − 0.04, *P* = 0.50).

adMNBIZ6 was positively associated with EGJ-RP (r = 0.15, *P* = 0.02), EGJ-RI (r = 0.16, *P* = 0.01), EGJ-CIG (r = 0.17, *P* < 0.01), adEGJ-CIG (r = 0.29, *P* < 0.0001), adEGJ-CIA (r = 0.19, *P* < 0.01), adEGJ-RP (r = 0.30, *P* < 0.0001), adEGJ-RI (r = 0.29, *P* < 0.0001) and DCI (r = 0.26, *P* < 0.0001). adMNBIZ6 was negatively associated with IEP (r = − 0.27, *P* < 0.0001), IGP (r = − 0.42, *P* < 0.0001), GEG (r = − 0.28, *P* < 0.0001) and EGJ-type (r = − 0.18, *P* < 0.01). adMNBIZ6 was not significantly associated with EGJ-P (r = 0.02, *P* = 0.77), EGJ-L (r = − 0.03, *P* = 0.60) and EGJ-CIA (r = − 0.004, *P* = 0.95). Spearman correlation analysis between esophageal HRM metrics and 24-h pH-impedance monitoring metrics was illustrated in Fig. S3 of Supplementary materials.

### Logistic regression analysis of independent risk factors for GERD

The original measured metrics of HRM and MNBI were included into Logistic regression because the other metrics were derived from the calculation or a combination of the original ones. Repeated including the same metrics irrespective of any kinds of transformation impairs the validity of Logistic regression. Moreover, the classification between GERD and non-GERD was directly based on AET, so AET was also not included in Logistic regression. Logistic regression showed that higher BMI and higher reflux episodes were independent risk factors for GERD development as shown in Wald plot and forest plot. Logistic regression also showed that male and higher MNBIZ6 were protective factors in GERD development. Wald plot and forest plot were illustrated in Fig. S4 of Supplementary materials.

### DCA of the clinical net benefit of esophageal HRM metrics and MNBI

**Figure 4:**
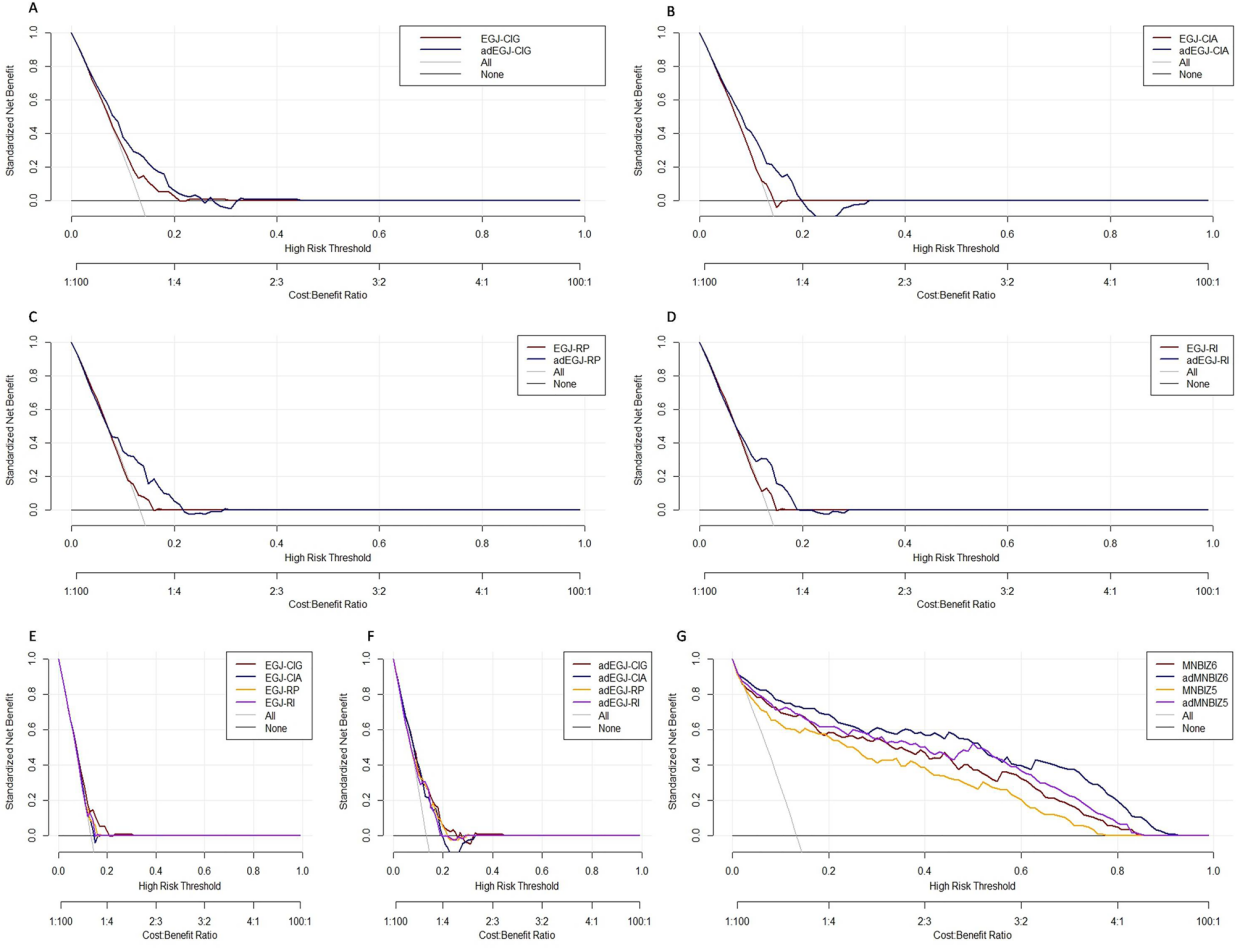
Decision curve of esophageal HRM metrics and MNBI in predicting GERD susceptibility. (**A**) Decision curve of EGJ-CIG and adEGJ-CIG in predicting GERD susceptibility. (**B**) Decision curve of EGJ-CIA and adEGJ-CIA in predicting GERD susceptibility. (**C**) Decision curve of EGJ-RP and adEGJ-RP in predicting GERD susceptibility. (**D**) Decision curve of EGJ-RI and adEGJ-RI in predicting GERD susceptibility. (**E**) Decision curve of EGJ-CIG, EGJ-CIA, EGJ-RP and EGJ-RI in predicting GERD susceptibility. (**F**) Decision curve of adEGJ-CIG, adEGJ-CIA, adEGJ-RP and adEGJ-RI in predicting GERD susceptibility. (**G**) Decision curve of MNBIZ6, MNBIZ5, adMNBIZ6 and adMNBIZ5 in predicting GERD susceptibility. *EGJ*-*RP* EGJ retention pressure, *EGJ*-*RI* EGJ-RP integral, *EGJ*-*CIA* EGJ contractile integral (EGJ-CI) by the reference to atmospheric pressure, *EGJ*-*CIG* EGJ-CI by the reference to intra-gastric pressure, *adEGJ*-*CIA* EGJ-CIA adjusted with BMI, *adEGJ*-*CIG* EGJ-CIG adjusted with BMI, *adEGJ*-*RP* EGJ-RP adjusted with BMI, *adEGJ*-*RI* EGJ-RI adjusted with BMI, *MNBIZ5* mean nocturnal baseline impedance channel 5, *MNBIZ6* mean nocturnal baseline impedance channel 6, *adMNBIZ5* MNBIZ5 adjusted with BMI, *adMNBIZ6* MNBIZ6 adjusted with BMI.

The prevalence of GERD was derived from a meta-analysis^1^. And 13.3% of the prevalence of GERD was employed to perform DCA in our study^1^. DCA showed that the clinical net benefit of EGJ-CIG was superior to that of EGJ-CIA, EGJ-RP and EGJ-RI when the above metrics were used to guide initiating treatment. The clinical net benefit of adEGJ-CIG, adEGJ-CIA, adEGJ-RP and adEGJ-RI was superior to that of the original ones. However, the high risk threshold range of the esophageal HRM metrics including EGJ-CIG and adEGJ-CIG was not very wide. DCA of the esophageal HRM metrics was illustrated in Fig. 4A–F.

DCA showed that the clinical net benefit of MNBIZ6 was superior to that of MNBIZ5 when the above metrics were used to guide initiating treatment. The clinical net benefit of adMNBIZ5 and adMNBIZ6 was superior to that of the original ones. Moreover, the high risk threshold range of MNBI was very wide. DCA of MNBI was illustrated in Fig. 4G.

## Discussion

This study explores the pathophysiology of GERD and has demonstrated that BMI is an independent risk factor for GERD. We incorporated BMI into the calculations of EGJ-CIG and distal MNBI, and improved their ability to discriminate GERD from non-GERD. We also designed novel HRM metrics to discriminate GERD and found that the novel HRM metrics was not superior to EGJ-CIG.

Direct comparison showed that EGJ-P was lower in the GERD group, and EGJ-L was shorter in the GERD group. However, the results did not reach statistical significance. Previous studies also showed that LES rest pressure was not significantly different between GERD and non-GERD^6,21^. This indicates that changes of EGJ-P or EGJ-L do not independently contribute to the development of GERD. Moreover, previous studies found that IGP and GEG were increased in GERD^6,22^. Our study has found a similar phenomenon. One study also indicates that IGP is associated with GERD after sleeve gastrectomy^23^. Therefore, IGP and GEG seem to be the main factors contributing to the development of GERD. HRM metrics do not exist independently but are with interactions. Our study showed that EGJ-P was positively correlated with IGP, which indicates that increased EGJ-P might be an adaptive response to increased IGP. This adaptive response has a role in anti-reflux in the condition of increased IGP. Subgroup analysis showed that IGP was positively correlated with EGJ-P in participants with EGJ type 1 and type 2, but not in participants with EGJ type 3. So the adaptive response of EGJ-P to increased IGP is impaired in participants with EGJ Type 3. EGJ Type 3 is a definite sign of esophageal hiatal hernia^9^. Hiatal hernia diminishes the length of abdominal esophagus and impairs the normal function of the flap valve of the gastric cardia^24,25^. So IGP is likely to act on abdominal esophagus and flap valve of gastric cardia to increase EGJ-P to prevent reflux when the flap valve of the gastric cardia is intact. Our study also found that IEP was increased in GERD as compared with that in non-GERD, and IGP was positively correlated with IEP. The explanation might be that increased IGP exceeds the threshold of EGJ-P adaptive response, and a part of IGP would be transmitted through EGJ and consequently increase IEP. So increased IEP found in our study is not the cause of GERD but rather the result of GERD.

Obesity is positively associated with GERD symptoms and esophageal acid burdens^10^. Our study showed that BMI was significantly higher in GERD as compared with that in non-GERD. Correlation analysis indicated that BMI was correlated with AET, IEP, IGP, and GEG positively. Logistic regression including esophageal HRM metrics also showed BMI as an independent risk factor for GERD. However, Spearman correlation analysis showed that BMI was not significantly associated with EGJ-P. Moreover, IGP was positively correlated with EGJ-P and IEP. Taken together, obesity might first increase IGP, and then IGP exceeds the adaptive response of EGJ-P and subsequently causes gastric reflux which causes IEP to increase. Another explanation is that increased abdominal fat causes the diaphragm to elevate and makes pulmonary expansion diminish, and elevated diaphragm would increase intra-thoracic pressure and IEP^26^. So IGP not IEP plays an important role in the GERD development in obesity.

Chicago Classification version 4.0 recommends EGJ-CI to be referenced to intra-gastric pressure instead of atmospheric pressure^9^. Our study found that EGJ-CIA was lower in GERD than that in non-GERD, but the difference did not reach significance. ROC showed that EGJ-CIG was superior to EGJ-CIA in discriminating GERD from non-GERD. These results support the recommendation of EGJ-CIG instead of EGJ-CIA by Chicago Classification version 4.0 to be used to discriminate GERD from non-GERD. We designed novel esophageal HRM metrics including EGJ-RP and EGJ-RI to discriminate GERD from non-GERD. However, these novel esophageal HRM metrics are not superior to EGJ-CIG in discriminating GERD from non-GERD. This implies that IEP is not the main contributor to GERD development in the general population, and incorporating IEP into the calculation of novel HRM metrics would not provide additional value to predict GERD susceptibility. BMI is associated with AET and multiple esophageal HRM metrics, and BMI is an independent risk factor for GERD. So we incorporate BMI into calculations of interested esophageal HRM metrics. As bigger BMI renders people to develop GERD, so EGJ-CI, EGJ-RP and EGJ-RI were divided by standardized BMI to obtain BMI adjusted EGJ-CI (adEGJ-CI), BMI adjusted EGJ-RP (adEGJ-RP) and BMI adjusted EGJ-RI (adEGJ-RI). The results showed that this method increased their ability to predict GERD susceptibility.

Different from esophageal HRM which measures motility in GERD, MNBI measures the mucosal damage caused by reflux contents. Esophageal baseline impedance reflects esophageal mucosal integrity^15^. Esophageal mucosal barrier composed of non-keratinized squamous epithelium with tight junctions is important in preventing mucosa from damages caused by gastric reflux contents^27^. Gastric reflux contents cause transepithelial electrical resistance to diminish and intercellular spaces of mucosa to dilate, and transepithelial electrical resistance can be detected by the change of nocturnal baseline impedance during 24-h pH-impedance monitoring^28–31^. Our study showed that MNBI of distal channels was significantly lower in GERD than in non-GERD. A previous study has proven the ability of MNBIZ6 to discriminate GERD from non-GERD^16^. Our study proved the diagnostic value of MNBIZ6 in GERD once more, and the cutoff was very close to the recommended one in Lyon consensus. Our study also indicated that BMI was negatively associated with MNBI of distal channels. The mechanism might be that esophageal squamous epithelial intercellular space diameter increases in obesity^32^. Logistic regression including MNBI variables also showed BMI as an independent risk factor for GERD. This is supported by a previous study with similar results^17^. As BMI is an independent risk factor for GERD, and bigger BMI renders people to develop GERD, so MNBIZ5 and MNBIZ6 were divided by standardized BMI to obtain BMI adjusted MNBIZ5 (adMNBIZ5) and BMI adjusted MNBIZ6 (adMNBIZ6).We found that this method improved the discriminating efficacy of MNBI for GERD.

Our study also indicated significant associations among AET, distal channel MNBI and esophageal HRM metrics. The results showed that adEGJ-CIG was the factor most correlated with AET among esophageal HRM metrics, which supported adEGJ-CIG to be used for evaluation of GERD susceptibility. Spearman correlation analysis also showed that IGP was the factor most correlated with MNBIZ6 among esophageal HRM metrics in our study. As MNBIZ6 represents esophageal mucosal integrity, IGP might be a risk factor for GERD related esophageal mucosal integrity impairment. This implies that decreasing IGP might be important for the avoidance of mucosal damages in clinical settings. BMI is correlated with IGP, so weight loss, especially abdominal weight loss might protect the esophageal mucosa through decreasing IGP. All of the above support weight loss as a therapy for GERD and GERD related esophageal mucosal damages.

Our study used DCA to assess the clinical net benefit of interested metrics. DCA guiding GERD treatment is different from other diseases with high mortality rates. The results indicate that EGJ-CIG and adEGJ-CIG are useful to discriminate GERD from non-GERD in clinical settings. However, the high risk threshold is not very wide. The net benefit of EGJ-CIG and adEGJ-CIG is warranted only when the high risk threshold is between 0.02 and 0.21. MNBIZ5, MNBIZ6, adMNBIZ6 and adMNBIZ5 are also useful to discriminate GERD from non-GERD in clinical settings. Moreover, the high risk threshold is very wide. The net benefit of MNBIZ5, MNBIZ6, adMNBIZ6 and adMNBIZ5 is warranted when the high risk threshold is 0.03–0.77. These implicate that EGJ-CIG and adEGJ-CIG can be used to confirm the susceptibility of GERD and add more valuable information to guide treatment when the probability of GERD is great. However, the value of EGJ-CIG and adEGJ-CIG is limited when the probability of GERD is very low. In the real world clinical settings, GERD questionnaire (GerdQ) and proton pump inhibitor (PPI) trial can be used to assess the probability of GERD, and guide the optimal utilization of EGJ-CIG and adEGJ-CIG. Correspondingly, MNBIZ5, MNBIZ6, adMNBIZ6 and adMNBIZ5 can be used both to confirm and exclude the susceptibility of GERD in a wide range of probability of GERD.

This study has its own merits. First, as age and gender can influence the development of GERD^2,17^, so age and gender are confounding factors in most studies evaluating GERD susceptibility and treatment. To eliminate the confounding effect of age and gender is of paramount importance to a successful study. Our study used PSM to balance the distribution of age and gender between groups. With the evaluation of propensity score distribution between groups before and after PSM, we found PSM made age and gender balanced between groups. So the confounding effect of age and gender is eliminated with this method. Studies with balanced groups can provide valid conclusions most close to the truth. Moreover, over matching should be avoided. Because BMI and other parameters are study variables in our study, these parameters were not used to perform PSM. Second, we used a sample size of controls more than three times bigger than that of cases in the original data to do PSM. This can make PSM more efficient to match cases and controls strictly. Moreover, case group and control group have the same sample size after PSM, which makes ROC work more efficiently to detect differences among interested metrics. Third, data of definite GERD and definite non-GERD with GERD symptoms is used in our study. This study design makes ROC more accurate to calculate true positive (TP), true negative (TN), false positive (FP) and false negative (FN), and provides more accurate sensitivity and specificity of interested metrics. Forth, as we know, this study is the first one using BMI to adjust EGJ-CI and MNBI in discriminating GERD from non-GERD, and it is proved by our study that BMI adjusted EGJ-CI and MNBI are superior to the original ones. As obesity is very common worldwide^33^, adjusting EGJ-CI and MNBI with BMI has great significance in clinical settings.

Our study has limitations. First, our study is a retrospective one based on available data. Some other interested variables including severity of esophagitis, BE and GerdQ are not included into our analysis. Second, data of treatment response are not retrieved. The effect of esophageal HRM metrics and MNBI on treatment response of GERD is not evaluated in this study. Third, due to the study design, we did not include borderline participants with AET between 4 and 6% defined by Lyon consensus^18^. The diagnostic value of interested metrics for discriminating this group of people is not evaluated in this study. Forth, the outcome of GERD patients with antireflux surgery (ARS) was lacking in this study, we cannot evaluate the correlation between HRM metrics and the outcome of ARS. Whether the HRM metrics of our study can guide ARS needs to be elucidated in the future studies. Fifth, our study was lack of the data of neural signal evaluation and esophageal pathology, whether a neural response and esophageal transmural inflammation participate in the adaptive response of EGJ-P to increased IGP also needs to be studied in the future studies. Sixth, due to strict screening of participants, the sample size of our study is not very large. More prospective randomized controlled studies with a large sample size or real world big data studies are needed to confirm the conclusion drawn from this study.

In conclusion, according to our study, EGJ-P do not independently contribute to the development of GERD, IGP and GEG might be the main factors contributing to the development of GERD. IGP might affect IEP through the inadequate adaptive response of EGJ-P to IGP. Increased IEP is likely not the cause of GERD but rather the result of GERD. Obesity causes IEP to increase via elevated IGP. And IGP not IEP plays an important role in the GERD development in obesity. EGJ-CI should be referenced to intra-gastric pressure instead of atmospheric pressure in determining GERD susceptibility. BMI is an independent risk factor for GERD. BMI should be considered when EGJ-CI and MNBI are used to predict GERD susceptibility. Incorporating BMI into the calculations of EGJ-CI and MNBI can improve their ability in predicting GERD susceptibility.

### Supplementary Information

Supplementary Information.

## Data Availability

The data that supports the findings of this study are available from the corresponding author upon reasonable requests.
