# Supplementary material for: Incorporating body mass index into esophageal manometry metrics and mean nocturnal baseline impedance for the evaluation of gastro-esophageal reflux disease
Source: Sci Rep. 2024 Aug 6;14:18269. doi: 10.1038/s41598-024-69253-2 (PMC11303378; doi:10.1038/s41598-024-69253-2)
Supplement: Supplementary file 1 — Supplementary Information. [file 41598_2024_69253_MOESM1_ESM.docx]

**Table S1.** The distribution of age and gender between the groups before and after PSM.

|  | Before PSM | | | After PSM | | |
| --- | --- | --- | --- | --- | --- | --- |
|  | GERD | Non-GERD | *P* | GERD | Non-GERD | *P* |
| Age (years)* | 60(46-68) | 54(40-64) | <0.01 | 60 (46-68) | 60 (47-67) | 0.93 |
| Gender (Male/Female) | 71/63 | 176/333 | <0.001 | 71/63 | 66/68 | 0.54 |

*Data was expressed with median, 25th percentile and 75 percentile. Abbreviations: PSM, propensity score matching.

**Table S2.** ROC comparison among esophageal HRM metrics in determining GERD susceptibility.

|  | EGJ-RP | EGJ-RI | EGJ-CIA | EGJ-CIG | adEGJ-CIA | adEGJ-CIG | adEGJ-RP |
| --- | --- | --- | --- | --- | --- | --- | --- |
| EGJ-RI | *P*=0.44 |  |  |  |  |  |  |
| EGJ-CIA | *P*=0.20 | *P*=0.09 |  |  |  |  |  |
| EGJ-CIG | *P*=0.34 | *P*=0.59 | *P*<0.001 |  |  |  |  |
| adEGJ-CIA | *P*=0.03 | *P*=0.06 | *P*<0.0001 | *P*=0.03 |  |  |  |
| adEGJ-CIG | *P*<0.001 | *P*<0.01 | *P*<0.0001 | *P*<0.0001 | *P*=0.16 |  |  |
| adEGJ-RP | *P*<0.0001 | *P*<0.0001 | *P*<0.001 | *P*=0.047 | *P*=0.72 | *P*=0.57 |  |
| adEGJ-RI | *P*<0.0001 | *P*<0.0001 | *P*<0.001 | *P*=0.03 | *P*=0.57 | *P*=0.73 | *P*=0.64 |
|  |  |  |  |  |  |  |  |

Abbreviations: EGJ-RP, EGJ retention pressure; EGJ-RI, EGJ-RP integral; EGJ-CIA, EGJ contractile integral (EGJ-CI) by the reference to atmospheric pressure; EGJ-CIG, EGJ-CI by the reference to intra-gastric pressure; adEGJ-CIA, EGJ-CIA adjusted with BMI; adEGJ-CIG, EGJ-CIG adjusted with BMI; adEGJ-RP, EGJ-RP adjusted with BMI; adEGJ-RI, EGJ-RI adjusted with BMI.

**Table S3.** ROC comparison among MNBI and MNBI adjusted with BMI in determining GERD susceptibility.

|  | MNBIZ5 | MNBIZ6 | adMNBIZ5 |
| --- | --- | --- | --- |
| MNBIZ6 | *P*<0.01 |  |  |
| adMNBIZ5 | *P*<0.0001 | *P*=0.11 |  |
| adMNBIZ6 | *P*<0.0001 | *P*<0.001 | *P*=0.02 |

Abbreviations: MNBIZ5, mean nocturnal baseline impedance channel Z5; MNBIZ6, mean nocturnal baseline impedance channel Z6; adMNBIZ5, MNBIZ5 adjusted with BMI; adMNBIZ6, MNBIZ6 adjusted with BMI.


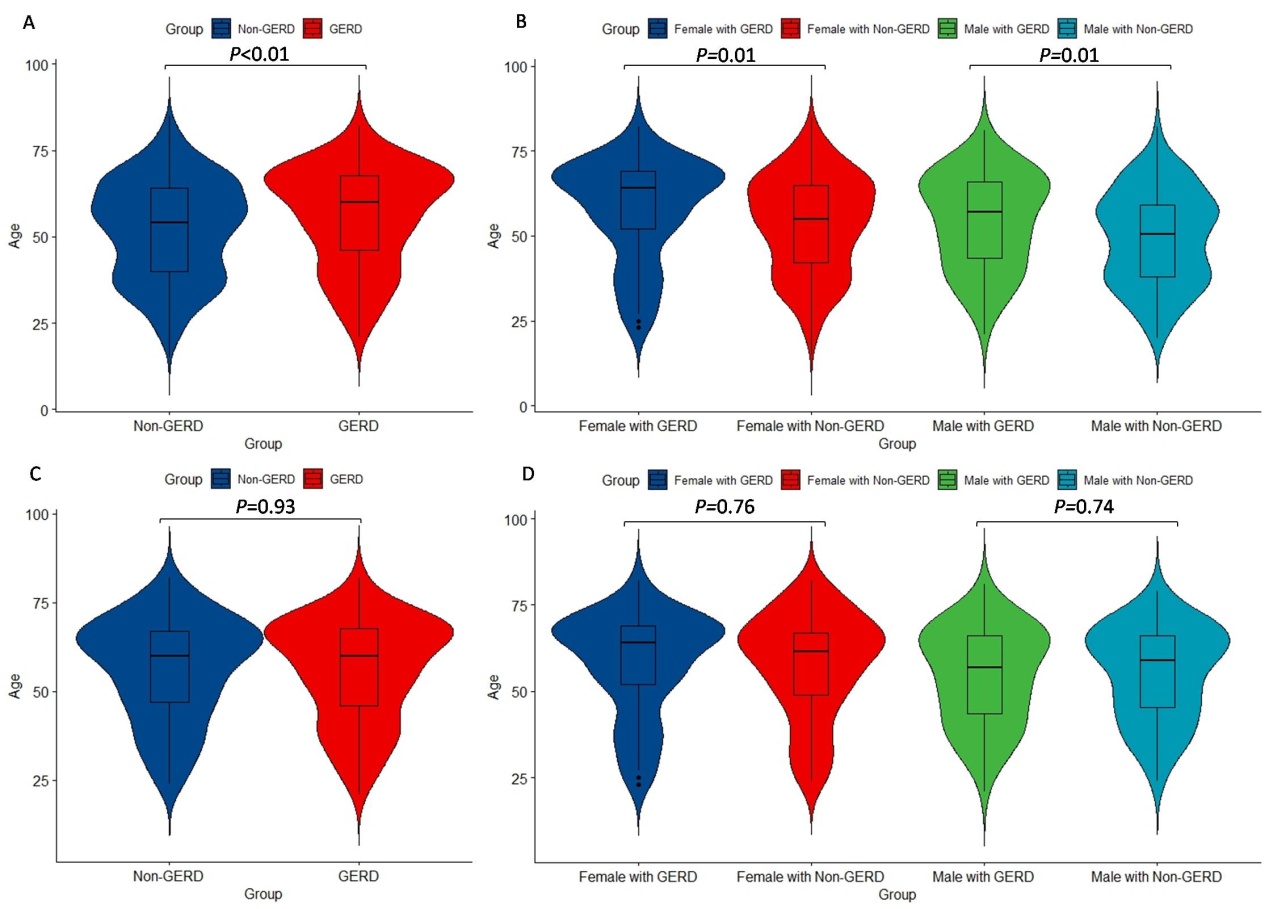


**Figure S1.** The distribution of age before and after PSM. (A) The distribution of age in the GERD group and the Non-GERD group before PSM. (B) The distribution of age according to gender in the GERD group and the Non-GERD group before PSM. (C) The distribution of age in the GERD group and the Non-GERD group after PSM. (D) The distribution of age according to gender in the GERD group and the Non-GERD group after PSM. Abbreviations: PSM, propensity score matching.


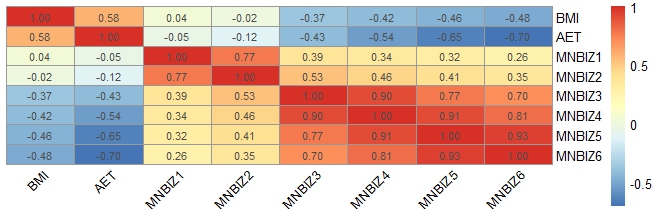


**Figure S2.** Spearman correlation analysis of BMI and MNBI. Abbreviations: BMI, body mass index; AET, acid exposure time; MNBIZ1-Z6, mean nocturnal baseline impedance channel Z1-Z6.


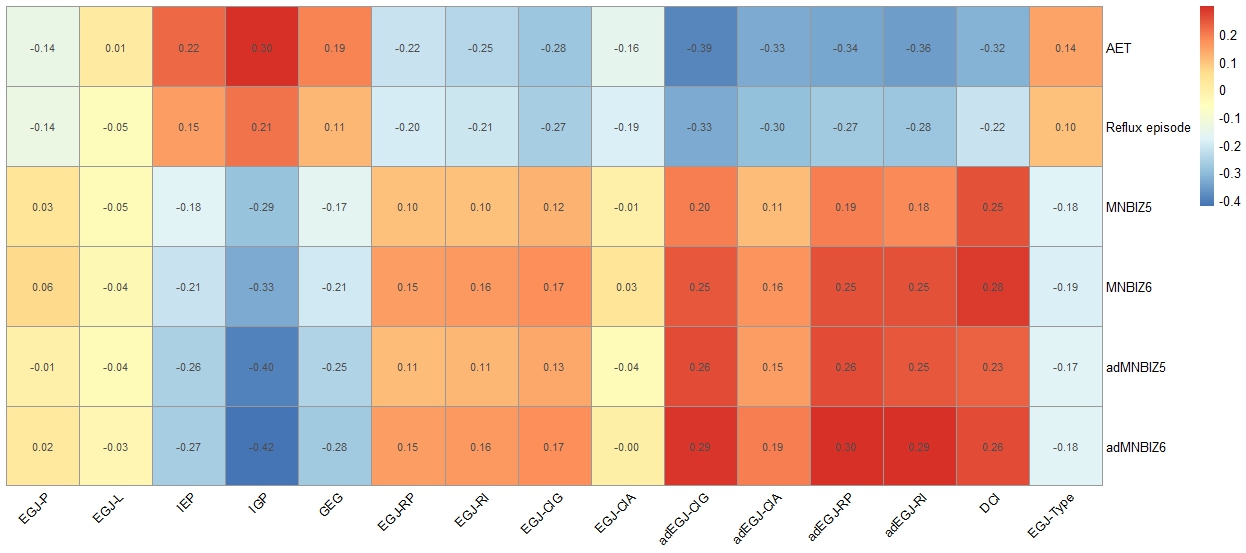


**Figure S3.** Spearman correlation analysis between HRM and pH-impedance monitoring metrics. Abbreviations: MNBIZ5, mean nocturnal baseline impedance channel Z5; MNBIZ6, mean nocturnal baseline impedance channel Z6; AET, acid exposure time; EGJ-P, EGJ rest pressure; EGJ-L, EGJ length; IEP, intra-esophageal pressure; IGP, intra-gastric pressure; GEG, gastro-esophageal pressure gradient; EGJ-RP, EGJ retention pressure; EGJ-RI, EGJ-RP integral; EGJ-CIG, EGJ contractile integral (EGJ-CI) by the reference to intra-gastric pressure; EGJ-CIA, EGJ-CI by the reference to atmospheric pressure; DCI, distal contractile integral; adEGJ-CIA, EGJ-CIA adjusted with BMI; adEGJ-CIG, EGJ-CIG adjusted with BMI; adEGJ-RP, EGJ-RP adjusted with BMI; adEGJ-RI, EGJ-RI adjusted with BMI.


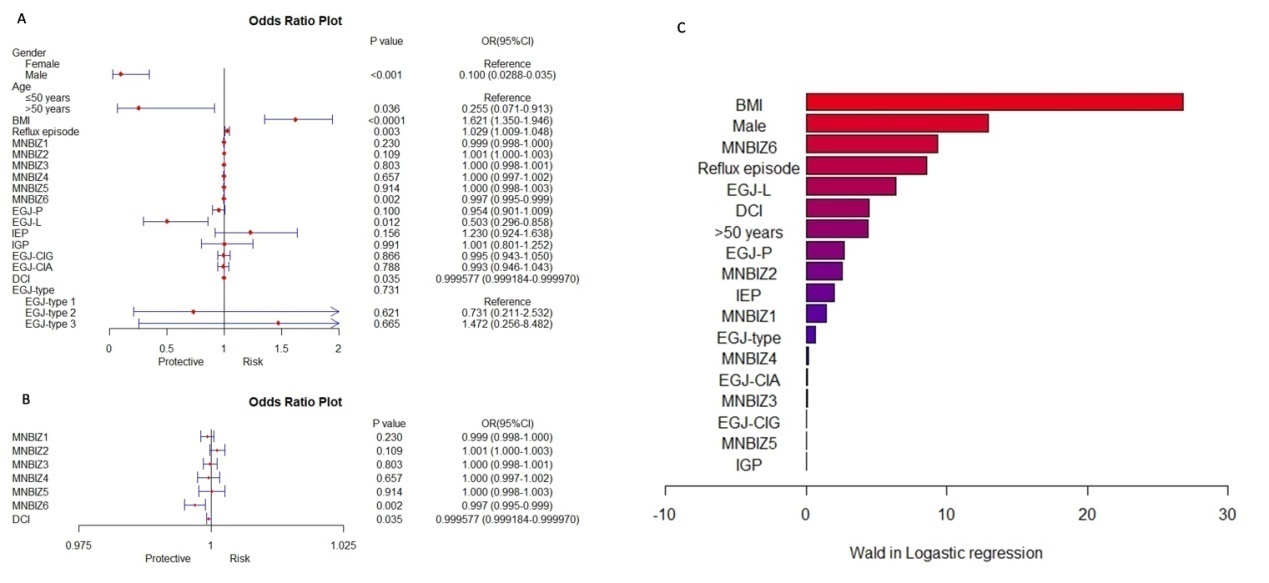


**Figure S4.** Forest plot and Wald distribution in Logistic regression of risk factors for GERD. (A) Forest plot of Logistic regression of all risk factors for GERD. (B) Forest plot of Logistic regression of risk factors with narrow 95%CI of OR for GERD. (C) Wald in Logistic regression of risk factors for GERD. Abbreviations: OR, odds ratio; 95%CI, 95% confidence interval; BMI, body mass index; EGJ-P, EGJ rest pressure; EGJ-L, EGJ length; DCI, distal contractile integral; IEP, intra-esophageal pressure; IGP, intra-gastric pressure; EGJ-CIA, EGJ contractile integral (EGJ-CI) by the reference to atmospheric pressure; EGJ-CIG, EGJ-CI by the reference to intra-gastric pressure; MNBIZ1, mean nocturnal baseline impedance channel Z1; MNBIZ2, mean nocturnal baseline impedance channel Z2; MNBIZ3, mean nocturnal baseline impedance channel Z3; MNBIZ4, mean nocturnal baseline impedance channel Z4; MNBIZ5, mean nocturnal baseline impedance channel Z5; MNBIZ6, mean nocturnal baseline impedance channel Z6.
